# Yearning to Give Back: Searching for Social Purpose in Computer Science and Engineering

**DOI:** 10.3389/fpsyg.2017.01178

**Published:** 2017-07-18

**Authors:** Coleen M. Carrigan

**Affiliations:** Social Sciences, California Polytechnic State University San Luis Obispo, CA, United States

**Keywords:** gender, computer science and engineering, social purpose, feminism, ethnography, social/technical divide

## Abstract

Computing is highly segregated and stratified by gender. While there is abundant scholarship investigating this problem, emerging evidence suggests that a hierarchy of value exists between the social and technical dimensions of Computer Science and Engineering (CSE) and this plays a role in the underrepresentation of women in the field. This ethnographic study of women's experiences in computing offers evidence of a systemic preference for the technical dimensions of computing over the social and a correlation between gender and social aspirations. Additionally, it suggests there is a gap between the exaltation of computing's social contributions and the realities of them. My participants expressed a yearning to contribute to the collective well-being of society using their computing skills. I trace moments of rupture in my participants' stories, moments when they felt these aspirations were in conflict with the cultural values in their organizations. I interpret these ruptures within a consideration of yearning, a need my participants had to contribute meaningfully to society that remained unfulfilled. The yearning to align one's altruistic values with one's careers aspirations in CSE illuminates an area for greater exploration on the path to realizing gender equity in computing. I argue that before a case can be made that careers in computing do indeed contribute to social and civil engagements, we must first address the meaning of the social within the values, ideologies and practices of CSE institutions and next, develop ways to measure and evaluate the field's contributions to society.

## Introduction: methods and frameworks

Computing knowledge is produced in highly segregated classrooms, labs, and workplaces, and many of these sites are rife with exclusionary practices (Cohen and Swim, 1995; Margolis and Fisher, 2002; Barker et al., 2005; Misa, 2011; Corbett and Hill, 2015). To understand this problem, I conducted a 2-year ethnographic study exploring several domains of women engineers' experiences in computing institutions. Main findings warrant further attention, including the role social aspirations play in the underrepresentation of women in Computer Science and Engineering (CSE). The data I present in this paper is a subsample of a large, complex dataset. It augments other qualitative studies that support connections between altruism, computing, and women (Hacker, 1981; Faulkner, 2000a,b; Margolis et al., 2000; Cuny and Aspray, 2001; Margolis and Fisher, 2002) and more recent quantitative studies that show similar results (Diekman et al., 2010, 2016; Cech, 2013b, 2014; Garibay, 2015; Litchfield and Javernick-Will, 2015; Blanchard Kyte and Riegle-Crumb, 2017; Cheryan et al., 2017). These interdisciplinary studies advance the theory that social purpose is an important factor to consider in working toward gender equity in CSE. The data in this paper raises the question: if it is true that careers with social aspirations matter more to women more than men (Diekman et al., 2010; Canney and Bielefeldt, 2015; Blanchard Kyte and Riegle-Crumb, 2017), then *how might this knowledge best be used to desegregate computer science and engineering?* I argue this question needs careful consideration and more qualitative investigations to ascertain social values in this field. Central to this inquiry are issues regarding the social applications of computing, the hierarchy of value between the social and the technical, and the gap between the exaltation of computing's social contributions vs. their realities.

My ethnography relied on the lived experiences of 42 people who work as computer scientists and engineers in elite corporations and universities. Significant findings convey connections between social aspirations, computing, and gender that suggest future pathways for collective inquiry. With approval from my institutional review board for research with human subjects, I engaged participants across a range of sites, including conferences, workplaces, and university campuses (the majority of schools were research-intensive universities and polytechnics). I interviewed CSE students, university faculty, and computing knowledge workers in industry settings—both women (*n* = 38) and men (*n* = 4) from multiple racial/ethnic identities and sexualities. I chose this sampling strategy in order to solicit insights into computer technology from people who must navigate both privilege and marginalization to persist in their field.

I performed semi-structured interviews, focus group interviews, life history case studies, and participant observation in classrooms, computing workplaces, and technology conferences (Bernard, 2006; Spradley, 2016a,b). I analyzed my data using grounded theory techniques (Strauss and Corbin, 1997; Cohen et al., 2000). Grounded theory allowed me to prioritize emerging themes and ideas rather than merely verify existing claims. I interpreted my participants' descriptions of their experiences being female in sites of CSE production using Smith's (1987) concept of “rupture.” Rupture occurs in women's lives when they experience the tensions of being ruled by dominant group members while simultaneously a part of the ruling class.

I traced the concept of rupture in my participants' stories, via moments when they felt their personal aspirations in conflict with the cultural values of computing. I interpreted these stories within a consideration of yearning, a need to contribute meaningfully to society. Only female participants expressed this yearning. To explore women's desire to contribute across different social identities, I engaged hooks' concept of yearning: “the shared space and feeling [that] opens up the possibility of common ground where differences might meet and engage one another” (Hooks, 1990, p. 13). This understanding of how collective emotions challenge normative practices, behaviors, and values is a unifying force in social movements (Parker and Hackett, 2012; Dean, 2016) and may therefore prove useful in efforts to desegregate CSE.

## Yearning: “leaving the world a better place”

Too often, high aptitude girls and women ask: What are the social contributions of computer science and engineering? Many conclude it is a field detached from social and civil engagement and lacking community purpose. They are, therefore, inclined to take their talent elsewhere. For example, Olivia left her job as a software engineer at a renowned Fortune 50 tech company because she “yearned to give back.” She questioned how she was benefiting society and if the computing products she helped create were even benefitting customers. She asked:

What is the benefit of all this? There's *no social impact*. I'm just helping to make [the corporation] money. Helping the customer is not enough, because it's all about the bottom line—only [the corporation] benefits…I believe in the importance of giving back. It's a huge part of my story.

Because Olivia yearned to make social contributions but did not have the opportunity to do so in a computing corporation, she returned to university to earn a doctorate in Human Computer Interaction (HCI), a subfield in CSE that integrates the social and technical aspects of computing. By switching from software engineering to HCI, she persisted in the computing field while reconciling her work with her “altruistic identity” (Carlone and Johnson, 2007).

Becca, an early career programmer, also gravitated toward HCI. She used to believe CSE was “evil” until her faculty mentor, an expert in HCI, helped her see the social applications of programming:

Conducting gendered HCI research made me realize that there were some really cool things about computer science I had never thought of…it's not just syntax and debugging, it's real, you know—real applications that can make a difference.

By choosing a subfield with social applications, Olivia and Becca found ways to both persist in the CSE field while also giving back to society. Others were concerned with the field's lack of reputation for altruism. Lynn, a software developer at a high-tech corporation, struggled with the social purpose of work in CSE, specifically the public's perception of the field:

Lynn: Part of it is my fear that I'm doing something that I can't explain, and for some women that is appealing somehow.
Interviewer: What do you mean, you can't explain to lay people?
Lynn: Yeah, like explain to my mom what I do. In the field of medicine you can say, “I'm working on a cure for cancer,” or “I'm helping people,” and people might not know the details, they might not know the science behind it, but they understand the goal.
Interviewer: Right, right. Which is…?
Lynn: Some kind of social good, or leaving the world a better place…
Interviewer: So computer science doesn't have that?
Lynn: It does, it's just that it's not advertised; people don't know about it.

Lynn may be seeking to reconcile a normative gender identity with her labor in a nontraditional field (Foor and Walden, 2009). Perhaps this desire for one's social contribution to be recognized is why a significant number of participants in my 2-year study saw the biomedical field as a viable avenue to which they can contribute. Biomedicine in the US has “evolved out of tradition of service to suffering humanity” (Sobo and Loustaunau, 1997, p. 126) and thus may be at an advantage for attracting people who yearn to use their skills in service of the higher good. For example, Sylvia, a doctoral student in CSE, wants to use computer technology to enhance public health infrastructures. She explains:

Because that's kind of just who I am. But it's also my mentor, she always talked about “*You really need to do something that would affect everyone*, you don't want to just…write it in a paper and then nothing happens…*you need to apply it* and *you need to be helpful*.” That's why I've been working with the Public Health Department.

Sylvia not only yearns to enhance public welfare, she also had the encouragement from a trusted female mentor who overcame the challenges of the “double bind” (Malcom et al., 1976; Ong et al., 2011) to do the same. Sylvia's mentor gave heartfelt advice perhaps as a means of investing in her student's persistence and success in the CSE field.

Other participants, especially those in more senior positions, saw recruiting and mentoring women in computing as ways they could make social contributions. For example, Marina, senior leader in academic CSE, fulfilled her social change aspirations by encouraging and supporting other women in her field, and this work helped to mitigate symptoms of the “imposter syndrome:”

I will always live with the part of me that feels I'm a total failure no matter what I do. But I know that I can walk up to…say…Anna and I can tell Anna and I can make her feel better about herself: “You're capable of doing pretty much anything you want with your life. You are extraordinary.”

By advancing other women in computing, Marina feels she is doing her part to contribute to the world, quelling her own self-doubts. In this way, women's support of each other serves as both a recruiting and retention tool.

The difficulties of persisting as a marginalized community member may be mitigated if one feels that one's career is more than a personal drive for success but a cause for the greater good. Some of my participants in this study who yearned to make a difference persisted in CSE by concentrating in certain subfields where their technical skills have social application—specifically Human Computer Interaction (HCI), and public health and biomedicine—or by participating in gender equity practices in engineering education. On the one hand, that some areas of CSE provide opportunities for integrating practitioners' personal and professional commitments is promising. However, without further investigation of the *value of the social in CSE culture*, a culture that “continues to be driven by hegemonic masculinity,” we risk creating gender-segregated subfields within computing (Bystydzienski and Brown, 2012, p. 17).

## Probing the social/technical divide

CSE has tendencies to devalue and delegitimize socially applied knowledge, ideas, beliefs, and practices that are grounded in communal, people-oriented values (Faulkner, 2007; Hoh, 2009; Diekman et al., 2010) and can thwart the social aspirations of its practitioners (Cech, 2013a; Litchfield and Javernick-Will, 2015). Why does “engineering have a long history of casting aside social and humanistic knowledge” (Riley, 2014, p. 5)? Western society is governed by strict ideological binaries of public/private, male/female, logical/emotional, real/unreal, rationality/creativity, and the technical/social (Scheper-Hughes and Lock, 1987). Our technological society grants different valuation to each side of these binaries, privileging quantitative, abstract rationality, and subordinating social, material, qualitative inquiries of the world (Denzin et al., 2006; Foor and Walden, 2009; Richter and Paretti, 2009; Garibay, 2015). How can these cultural values and norms in computing—ones that denigrate the epistemic practices and findings of research with social applications (Diekman et al., 2010; Cech, 2013a)—be reconciled with the altruistic yearnings of its practitioners and widely accepted claims that computers promote human freedom and social advancement?

For example, Shawna, a doctoral student in CSE teamed up with an HCI professor to create applications for disabled computer users. In this collaboration, she not only faced resistance from her theory/algorithms advisor, but she also faced resistance from her peers:

Shawna: The lab itself was always a bit boisterous…they just really were pushing the technology, you know, and focusing more on the computer part, as opposed to the human part. And I felt that whenever I brought up the human issues, that they were ignored, mainly. In fact, I earned the moniker, “Accessibility Bitch.”
Interviewer: Oh, my God! That is so offensive.
Shawna: Yeah, actually, I took it as a compliment.
Interviewer: Really, even with the “B” word?
Shawna: Hey, I subscribe to the magazine.

Shawna's experiences evince the devaluation of the social in CSE. She uses humor to maintain confidence and pride in her efforts to use computers to support those with learning disabilities, despite the lack of support of her professor and hostility of her peers.

To leave epistemic injustices unexamined is a disservice to efforts to broaden participation in CSE fields. Diekman et al. (2010) suggest that an innovative way to attract more women to computing is to make it appear more socially applicable and in service of communal goals. To date, however, it appears that this promising solution may be thwarted by an endemic phenomenon in CSE culture that deems work with a social purpose to be irrelevant and outside the scope of an engineer's duties (Gilbert, 2008, 2009; Cech, 2014). Without interrogating the stubborn bond between masculinity and technology and confronting the epistemic bias that extols the technical at the expense of the social, we run the risk of creating pink-collar ghettos in CSE, subfields “labeled as inferior or “not real” engineering while male-dominated [sub]fields continue to garner social prestige and higher remuneration” (Bystydzienski and Brown, 2012, p. 4).

## Public service or public relations?

Before launching concerted public relations efforts to promote the social benefits of CSE and attract students with altruistic motivations, the question of *retaining* these types of students and workers needs further consideration. Not only is there evidence of gender and racial bias in computing (Corbett and Hill, 2015; Seron et al., 2015), there is also a considerable gap between what political and industry leaders say computers do for society and the true impacts of high-tech knowledge and applications (Mander, 1991; Hakken, 2003; Toyama, 2015).

This gap helps explain participants' uncertainty about whether or not CSE work provides opportunities to give back to society. For example, Lynn, the software developer quoted above, told me that she and one of her professors built an application for an eye-tracking device intended to help people with disabilities; however, this application was eventually utilized for marketing purposes in order “to track where people look on the screen for retrospective [marketing] analysis.” Her collaboration with this professor was a success and she was pleased that they published their findings. Lynn reflected that CSE professionals like the idea, or the *potential*, of applications for people with disabilities much more than the actual implementation of such applications:

At the time I wanted to do something for people with disabilities, and [my professors] liked the drawing program with the eyes because it was exciting and sexy, but when you start talking about, like, practical accessibility issues, people kind of turn off.

Lynn had explicitly said she wanted a career that others recognized as socially valuable. The eye-tracker project, which began as an effort to help people, ultimately morphed into a high-tech company's surveillance of customers. This experience contributed to Lynn's suspicion that the computing industry is altruistic more in theory than practice.

The social benefits of computing are a speculative promise that lack evidence. Popular discourse and corporate advertisements emphasize the positive impacts of technological innovations (and often, their *potential* social benefit alone) while eliding more insidious applications and outcomes. For example, computers enable a centralization of power that makes the world's population highly vulnerable to surveillance, unemployment, weaponry, objectification, and instrumentalized rationality that undermines civil liberties and civic engagement. Also, high-tech corporations are “philosophically antitax and it's decimating” the states in which they operate (Duhigg and Kocieniewski, 2012, p. 10). Despite these dangers, Toyama (2015) notes that computer technology has “a cult-like hold” on our society and admits that he feels disloyal in his critique of its purported contributions to society (p. xv).

Critical analyses of computing require risking suspicions of disloyalty. Given that large-scale computing work largely takes place in US industrial, commercial, or military domains (Pawley, 2012), social justice efforts in high-tech fields must question some of the most powerful institutions in the world. Change agents can push computer scientist and engineers not only to “welcome people on the margins,” but also to support local efforts of grassroots communities who are challenging hegemonic social relations like institutional racism, sexism, homophobia, classism, and environmental degradation (Pawley, 2012, p. 80). Capitalizing on the shared yearning of some CSE workers to contribute to the communal good may be a way to bridge computing's much-touted benevolentness and its actual outputs and impacts.

## Paths ahead

My participants' lives, careers, and aspirations not only challenge assumptions of who is allowed to participate in CSE work, but also the false binary between social and technical dimensions of computers. Before embarking on campaigns to laud the social benefits of computing knowledge and artifacts, let's check for evidence of these benefits first. This suggestion is not original. Probing the gap between what people *say* they do and what they *actually* do is one of the key tenets of cultural anthropological research (Guest, 2014) and the application of this methodology to scientists and engineers led to the field of science, technology, and society (STS) (Shapin et al., 1985; Franklin, 1995).

What evidence can change agents in CSE offer to convince skeptics that computing education is more than a boot camp for future employees of defense corporations and computing workplaces more than primary-colored playgrounds for a privileged few? How do we measure and evaluate the positive social contributions of CSE? Critiquing unjust practices within the field is a start. Combatting the disdain for socially relevant computing research in favor of technical knowledge and commodities is also necessary. Let's also questions the field's allegiances. For example, in its proprietary, classified form, computer knowledge production is a lucrative field that operates in tandem with state and corporate interests to erode community and social collectivity (Hakken, 2003; Coleman, 2013). What are the effects of collaborating with high-tech corporations in efforts to advance women in computing? Might we dare to question Bill Gates' claim that a laissez faire capitalist market and computing are inexorably entwined (Gates et al., 1995)?

Qualitative studies that examine the yearning of women in CSE across a range of cultural domains—race/ethnicity, sexuality, class, career stage, CSE subfields—may yield findings critical to broadening participation in this largely homogenous field. By examining structures of power through the lens of lived experience, we will not only deepen understandings of the cultural landscape of CSE, but also better determine what constitutes “good” science in the social/intellectual movement to end labor segregation in computer science and engineering.

## Conclusion

Although women's values certainly differ, my research suggests that some women value contributing to society using their technological savvy. More research is needed on the role of social purpose in working toward gender equity in CSE. Future research will require pulling back and looking at the broader picture of how computing is operating in and affecting our society. By paying attention to moments of rupture in the lives of my research participants, an emotional phenomenon—a yearning to give back—emerged. Some female CSE specialists experience conflict when they seek to align their altruistic values with their careers. How can women's attempts to reconcile their social and career aspirations in CSE generate further insight into segregation in the field and ways to combat this vexing problem? Systemic intervention cannot come to fruition without unbundling normative values regarding labor, gender identities, and computer technology. If we are going to promote the social impact of CSE work, a thorough assessment of what constitutes the social good will be required, as well as an invigoration of the imagination to envision computing in service of social justice.

## Ethics statement

This study was carried out in accordance with the recommendations of the University of Washington Institutional Review Board (IRB) for research with human subject's with written informed consent from all subjects. All subjects gave written informed consent in accordance with the Declaration of Helsinki. The protocol was approved by the the University of Washington IRB (#39150-EG).

## Author contributions

CC is the sole author of this manuscript and completed the research presented in this article independently, with the generous support of a American Association of University Women (AAUW) American Fellowship.

### Conflict of interest statement

The author declare that the research was conducted in the absence of any commercial or financial relationships that could be construed as a potential conflict of interest.
